# Multi-scale attention network (MSAN) for track circuits fault diagnosis

**DOI:** 10.1038/s41598-024-59711-2

**Published:** 2024-04-17

**Authors:** Weijie Tao, Xiaowei Li, Jianlei Liu, Zheng Li

**Affiliations:** 1 Department of Rail Transportation, Shandong Jiaotong University, Jinan, 250357 China; 2 Department of Cyberspace Security, Qufu Normal University, Jinan, 273165 China

**Keywords:** Multi-scale neural network, Gramian Angular Field (GAF), Spatial attention, Feature fusion, Fault diagnosis, Engineering, Mathematics and computing

## Abstract

As one of the three major outdoor components of the railroad signal system, the track circuit plays an important role in ensuring the safety and efficiency of train operation. Therefore, when a fault occurs, the cause of the fault needs to be found quickly and accurately and dealt with in a timely manner to avoid affecting the efficiency of train operation and the occurrence of safety accidents. This article proposes a fault diagnosis method based on multi-scale attention network, which uses Gramian Angular Field (GAF) to transform one-dimensional time series into two-dimensional images, making full use of the advantages of convolutional networks in processing image data. A new feature fusion training structure is designed to effectively train the model, fully extract features at different scales, and fusing spatial feature information through spatial attention mechanisms. Finally, experiments are conducted using real track circuit fault datasets, and the accuracy of fault diagnosis reaches 99.36%, and our model demonstrates better performance compared to classical and state-of-the-art models. And the ablation experiments verified that each module in the designed model plays a key role.

## Introduction

The rapid development of the railroad transportation industry attracts people's attention widely to the issue of safety and reliability of the railroad signaling system. Track circuit^1,2^ is one of the key equipments of railroad signaling system, which can ensure the safe and efficient operation of trains on the line. At present, ZPW-2000A track circuits are widely used in the railroad running intervals, the main function can be to check the interval idle and transmit information, the normal operation of its equipment is to ensure the safety of the train key. However, ZPW-2000A track circuits have many equipments installed outdoors, which are susceptible to failures affecting train safety and operational efficiency due to long-term environmental impacts. At present, the rail transit operation and management departments mainly use manual inspection, signal centralized monitoring and other measures to realize the fault diagnosis of uninsulated rail circuits. These methods mainly rely on the staff's own experience, which not only brings greater labor intensity and pressure to the staff, but also may cause diagnostic delays or diagnostic errors, seriously affecting the efficiency and safety of train operation^3^. When a fault occurs, it is necessary to quickly and accurately diagnose the type of fault in order to carry out timely repairs and restore the normal operation of the equipment. Therefore, the establishment of an efficient and accurate fault diagnosis model plays a crucial role in ensuring the safe operation of trains and improving the efficiency of train operation.

With the development of sensor technology, which can effectively and accurately collect large amounts of data, data-driven deep learning methods^4–6^ are widely used in feature extraction and have achieved remarkable results. Yang et al. proposed the use of a dual-input model based on multiscale convolution^7^ for fusing time–frequency domain features; The combination of CNN and LSTM proposed by Huang et al.^8^, where the CNN layer performs feature learning without relying on a priori knowledge and by using the LSTM layer, time delay information can be captured; Shang et al. constructed the Self-Attentive Gradient Penalized Wasserstein Generative Adversarial Network^9^ (SAWGAN-GP) to address the problems of poor quality of simulated data and training instability; Shao et al. proposed a multiscale deep convolutional neural network (MSD-CNN)^10^, which extracts the multiscale information of the original fault signal through multiscale cascaded convolutional kernels in the MSD-CNN. In the field of fault diagnosis about mechanical bearings, Huang et al. performed fault diagnosis in the case of incomplete training data, and proposed a framework based on cepstrum scale-distance for the incomplete training data^11^, which is more in line with the industrial status quo and can achieve good results. Ma et al. in response to the problem of the lack of rare fault samples in the course of the actual engineering training process. proposed a dynamic simulation model and gradient normalized Wasserstein Generative Adversarial Network (WGAN-GN) based on the simulation model to obtain the simulated signals^12^, and then use the augmented data in the WGAN-GN, and finally build a classification model based on the stacked autoencoder. Piltan et al. proposed a hybrid fuzzy V-structure fault estimator method for timely monitoring of bearing faults and identifying crack sizes, which combines a fuzzy algorithm and a V-structure approach to improve the estimation and prediction of unknown conditions^13^. Experimental results show that the average accuracy of fault classification and crack size identification using this method is 98.75% and 98%, respectively.

In the field of track circuit fault diagnosis, Zhao et al. established a track circuit simulation model to solve the problem of insufficient experimental data^14^, simulating different fault data for experiments; Chen et al. presented laboratory research results of track circuit fault monitoring and detection methods^15^.They combined a fuzzy system and a neural network to form a fault detection system, and experiments showed that the fuzzy neural network was effective in identifying various common fault patterns in orbital circuits; Lu et al. established a track circuit judgment model based on BP neural network^16^, which can realize the real-time judgment of track circuit signals. Bruin et al. proposed to establish a fault diagnosis method based on LSTM networks^17^, by combining spatial and temporal aspects of the original signal for fault diagnosis; Liu et al. proposed an adaptive fault diagnosis system for rail circuits^18^, which uses pattern recognition to extract fault features from simulation data. Dong et al. proposed to increase attribute reduction and adaptive use of genetic algorithm for fuzzy cognitive graph to pre-process fault data^19^. It can effectively diagnose faults in ZPW-2000A seamless track circuit; Zheng et al. proposed the use of an optimized particle swarm algorithm to optimize deep belief networks^20^, which improved the robustness and accuracy of the network; Lin et al. proposed a fault diagnosis method based on rough set and graph theory for ZPW-2000A uninsulated track circuits^21^, and proposed a new concept of fault decision chart for fault diagnosis.

Although the above methods provide different ideas and thoughts for diagnosing track circuit faults, there are still some unsolved problems. In some existing fault diagnosis methods, one-dimensional data is usually used, but when the data is processed, simple normalization or no data processing is usually used. This will have the problem that the model cannot mine the features in the data well^14–19^. Secondly, the fault diagnosis model does not consider feature extraction from different scales or dimensions, and the structure of the model is slightly lacking^20,21^, which affects the performance of the model.

The track circuit fault data we collected is one-dimensional time-series data, in order to fully utilize the advantages of CNN in image classification and recognition, so that the key features in the data can be extracted, we introduce the Gramian Angular Field (GAF) method to transform the one-dimensional raw data into two-dimensional images. The converted image data of this method retains the complete information of the signal and also maintains the dependence of the signal on time. In addition to this, we designed a multiscale attention network for diagnosing track circuit faults. The method constructs a feature fusion model based on multiscale convolutional network, U-Net network and spatial attention network, takes the image transformed by GAF as the input, extracts the features by multiscale attention network, and finally outputs the type of faults by a classifier. High-precision and efficient fault diagnosis is realized.

The main contributions of this paper can be summarized as follows:

1. In this paper, a GAF-based data preprocessing model is proposed to convert 1D time series data into image data by preprocessing. It retains the complete information of the signal as well as the dependence of the signal on time. The conversion of signal data into image data can then take full advantage of CNN in image classification recognition, which greatly improves the classification accuracy.
2. In this paper, we propose a feature fusion method. Firstly, the features at different scales are extracted by building a multi-scale convolutional network. Then by building a U-Net model, the low-resolution and high-resolution information in the original data is extracted to improve the classification accuracy. Finally, the features extracted by the two models are fused by building a spatial attention network. This method solves the problem of time-consuming and low efficiency of convolutional networks in the fault diagnosis process.
3. Fine-grained experiments were designed using real collected data sets, and the experimental results proved the usability and accuracy of the method, and showed the superiority of the method by comparing it with related methods.

The rest of this paper is organized as follows. Section "Related work" introduces related work. Section "Methods" describes the proposed method in detail. In Section "Experiments and results" a series of experiments are constructed and the experimental results and analysis are given. Finally, conclusions are drawn in Section "Discussion".

## Related work

Some methods attempt to achieve feature learning from data. Data-driven approaches^7–9^ are often based on machine learning algorithms. References^7,9^ apply multi-scale convolutional networks to mine feature information from raw data. Reference^8^ combines CNN and LSTM networks to learn temporal feature information. Reference^9^ uses a method that combines Wasserstein GAN network with self-attention network to address small-sample fault diagnosis problems.

Some methods have been proposed to deal with fault diagnosis problems in railway circuits. In^14^, Zhao et al. established a simulation model of the tuning unit for experimental simulation of fault data. However, this method only focuses on the tuning unit module in the railway circuit and does not involve faults of other equipment in the railway circuit. In^15^, Chen et al. extracted time–frequency features using wavelet transform. However, this method requires a large amount of training data to train the neural network, which may affect the effectiveness of the model if the data is insufficient. In^16^, the model is able to diagnose fewer types and needs further optimization. In^17^, only local feature parameters are considered, and the impact of global feature parameters on the diagnostic effect is not taken into account. Although the methods used in^17,18,20,21^ achieve better accuracy, they require a large amount of experimental data as support in practical applications, have a large computational load, and cannot be applied well in situations where there is a lack of large amounts of test data. Some methods can process raw data. In^19,20^, one-dimensional raw data is transformed into two-dimensional images using Gramian Angular Field (GAF), which preserves the temporal and feature information of the data. When processing image data, reference can be made to^28^ to use U-Net network to learn different levels of image features.

## Methods

**Figure 1:**
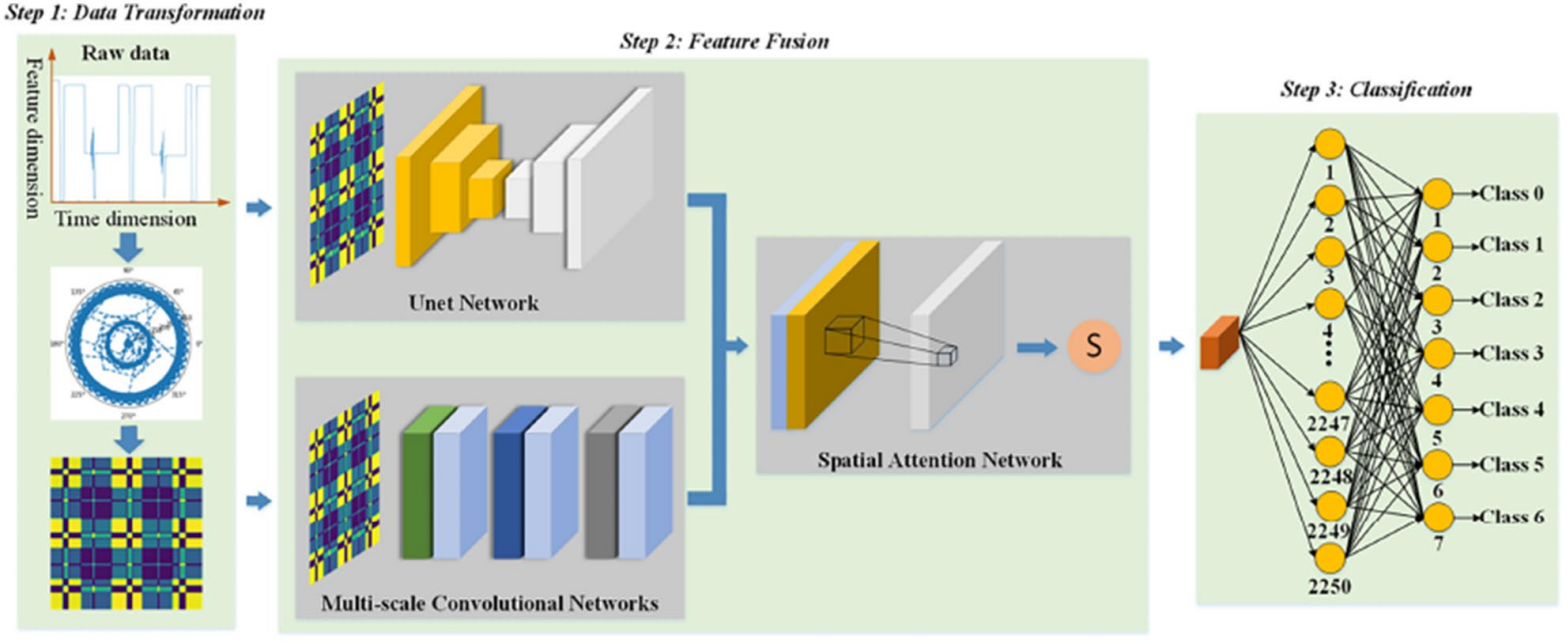
Overall framework diagram of MSAN.

In this paper, an effective fault diagnosis method for rail circuits is proposed, which is divided into three main steps, including data conversion, feature fusion and classification. Figure 1 shows the overall flow of the method.

### Gramian angular field (GAF)

This paper introduces the Gramian Angular Field (GAF)^22,23^ to transform one-dimensional raw data into two-dimensional pictures for two main reasons. One is that the fault diagnosis model is mainly built based on convolutional neural networks, and CNNs are the mainstream deep learning structures for processing image data, and they usually contain layers such as convolutional layers, pooling layers, and other layers used for processing two-dimensional image data. Converting one-dimensional data into two-dimensional images can make better use of these structures, thus giving full play to the advantages of convolutional neural networks in dealing with image classification tasks and realizing effective processing of image data. Secondly, the one-dimensional original data of the track circuit has a temporal correlation, and when using this method to process the data, not only can the one-dimensional data be transformed into two-dimensional pictures, but also can retain the original information of the data and the temporal correlation, so that no important information will be lost due to the transformation of the data.

This method can preserve the temporal correlations of one-dimensional data by converting scaled 1D sequential data from a Cartesian coordinate system to a polar coordinate system, and identifying temporal correlations at different time points by considering the angle summation or difference between different points. The advantage of using this method is that it not only preserves the temporal correlation of one-dimensional data, but also preserves the complete information of the data. The process of converting one-dimensional time series data to two-dimensional images using GAF is shown in the Step1 section of Fig. 1. The original one-dimensional data is used as input, transformed using GAF method and output as a two-dimensional image. The specific conversion process is shown below:

For a time series X = (xt,t = 1,2,…,N), first normalize the raw time series data to [0,1] by scaling.

1 $$ \mathop {\overset{\lower0.5em\hbox{$\smash{\scriptscriptstyle\smile}$}}{x} }\nolimits_{i} = \frac{{\mathop x\nolimits_{i} - \min (X))}}{\max (X) - \min (X)} $$

Then, transform the normalized data into a polar coordinate system to obtain the radius (r) and angle (Φ) corresponding to each data point.

2 $$ \left\{ {\begin{array}{*{20}c} {\varphi = \arccos (\overset{\lower0.5em\hbox{$\smash{\scriptscriptstyle\smile}$}}{x}_{i} ),} & { - 1 \le \overset{\lower0.5em\hbox{$\smash{\scriptscriptstyle\smile}$}}{x}_{i} \le \overset{\lower0.5em\hbox{$\smash{\scriptscriptstyle\smile}$}}{X} } \\ {{\text{r}} = \frac{{t_{i} }}{N},} & {t_{i} \in \overset{\lower0.5em\hbox{$\smash{\scriptscriptstyle\smile}$}}{X} } \\ \end{array} } \right. $$

Finally, use the angle-sum relationship to obtain the corresponding GASF diagram.

3 $$ \begin{aligned} GASF & = \left[ {\cos (\varphi_{i} + \varphi_{j} )} \right] \\ & = \overset{\lower0.5em\hbox{$\smash{\scriptscriptstyle\smile}$}}{X}^{\prime} \cdot \overset{\lower0.5em\hbox{$\smash{\scriptscriptstyle\smile}$}}{X} - \sqrt {I - \overset{\lower0.5em\hbox{$\smash{\scriptscriptstyle\smile}$}}{X}^{{\prime}{2}} } \cdot \sqrt {I - \overset{\lower0.5em\hbox{$\smash{\scriptscriptstyle\smile}$}}{X}^{2} } \\ \end{aligned} $$

### Feature fusion model

In order to better extract the features in the converted image, we constructed a feature fusion model, as shown in the Step 2 part of Fig. 1, which consists of a multi-scale convolutional neural network^24–26^, a U-Net network^27^ and a spatial attention network^28^. The feature fusion process first takes the two-dimensional images converted by GAF transformation as input, and extracts features separately using multi-scale convolutional neural network and U-Net network. Then, the extracted features are fused through a spatial attention network for feature fusion. This model obtains feature information of data at different scales and depths in the spatial dimension, improving the classification accuracy of the model and reducing training time.

**Figure 2:**
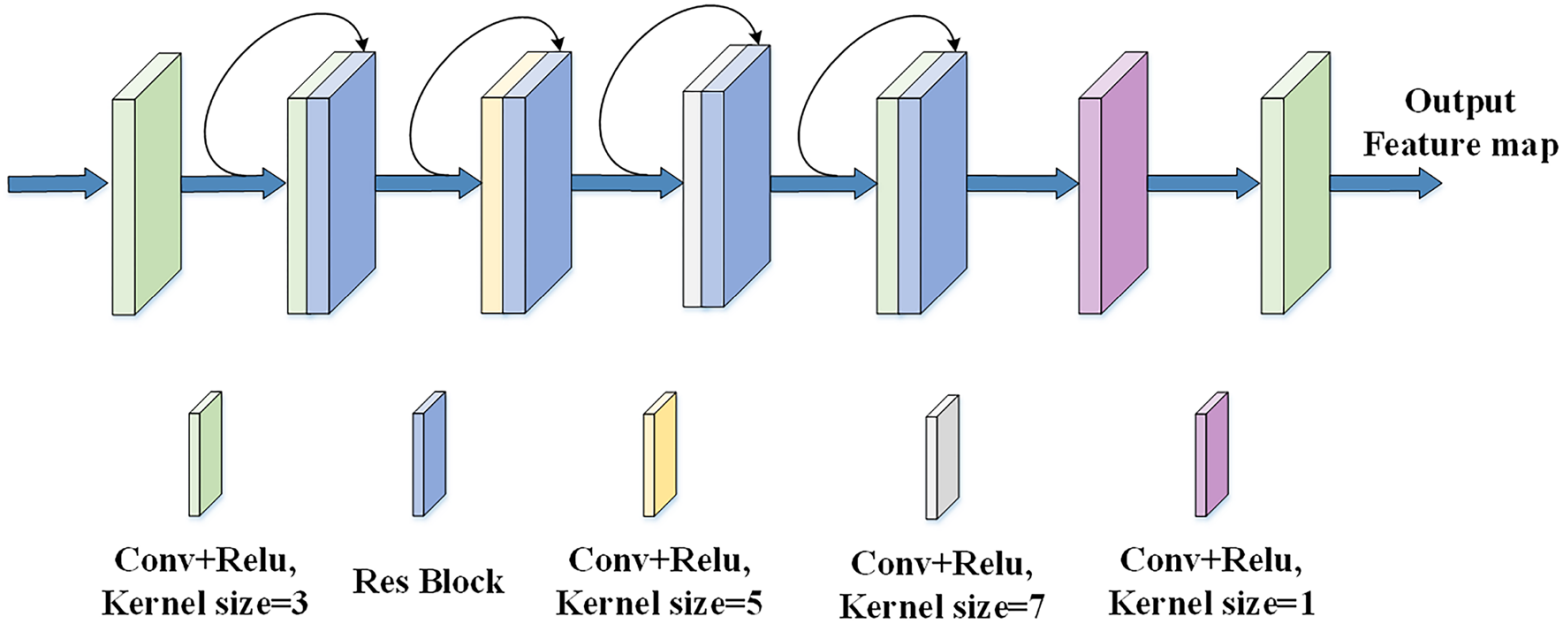
Multi-scale convolutional network architecture diagram.

The main architecture of the multiscale convolutional neural network is shown in Fig. 2.The multiscale convolutional neural network consists of seven convolutional layers. Among them, the green, yellow, gray and purple modules indicate the convolution and ReLU operation, and the size of the convolution kernel is 3*3, 5*5, 7*7 and 1*1 respectively.The blue module indicates the residual operation. The black curved arrow indicates a splice operation. The two-dimensional image processed by the GAF method has a scale of 64*64*3, which is used as input to the network. The input data first undergoes one convolution operation to obtain a 64*64*16 feature map. The feature map is then convolved once to obtain a second 64*64*16 feature map, and these two feature maps are spliced in the channel dimension and input into the residual block to obtain a 64*64*32 feature map. Repeat this step three times to get 64*64*80 feature maps, and finally after two convolutions to get 64*64*3 feature maps. In this process, we set different convolutional layers with different kernel sizes to better extract multi-scale feature maps and increase the number of feature maps. At the same time, residual blocks with different number of channels are set to increase the depth of the network.In this network, we set up convolutional layers with different convolutional kernel sizes and residual blocks with different numbers of channels, so that features of different scales and depths can be extracted efficiently.

**Figure 3:**
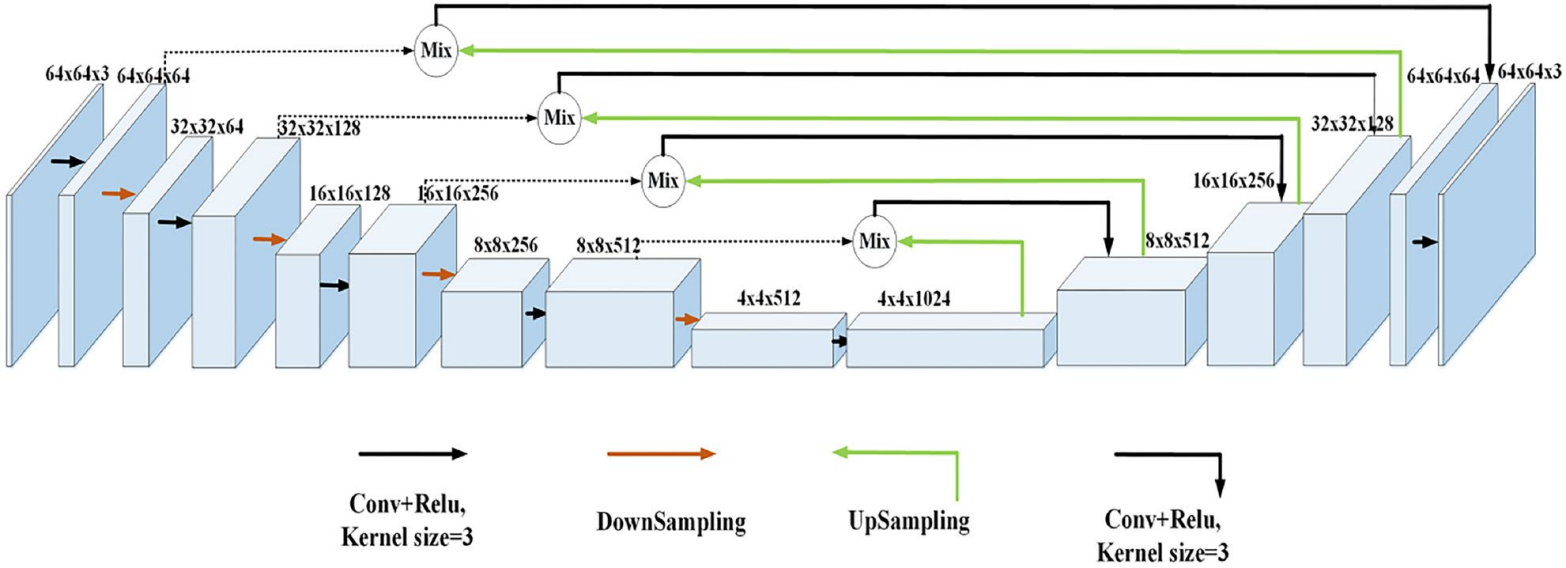
U-Net network architecture diagram.

The main architecture of the U-Net network is shown in Fig. 3, where the black solid arrows denote the convolution and ReLU operations, the black dashed arrows denote the splicing operation, the red arrows denote the downsampling operation, and the green arrows denote the upsampling operation. The left part is the encoding structure, which mainly extracts local features, continuously reduces the resolution of the feature map to capture context features. The encoder is divided into four stages. In each stage, different numbers of feature maps are obtained using a convolution operation with a 3 × 3 kernel size and stride of 1, and then downsampled using a convolution operation with a 3 × 3 kernel size and stride of 2. The right part is the decoding structure, which mainly performs feature fusion and restores the feature map to the original image size using the encoded features. The decoder is divided into four parts, symmetric to the encoder. Each stage consists of a deconvolution layer, feature concatenation, and a 3 × 3 convolution layer. Finally, a 1 × 1 convolution operation is performed to reduce the number of channels to a specific quantity. The GAF processed 2D image data is used as input to the U-Net network. The input image size is 64*64*3 and the output feature map size is 64*64*3. The variation of feature map in the network is shown in Fig. 3. The input 2D image data is first convolved once to adjust the number of channels of the feature map, then downsampled once to adjust the size of the feature map, and the process is repeated four times to obtain the final downsampled feature map. Then the final downsampled feature map is upsampled to expand the feature map size and reduce the number of channels. Then the feature map is spliced with the corresponding feature map on the left, and finally the number of channels is adjusted by convolution operation. This operation is repeated four times to restore the feature map to the original image size and number of channels. The contextual feature information at different scales can be effectively captured by the U-Net network.

**Figure 4:**
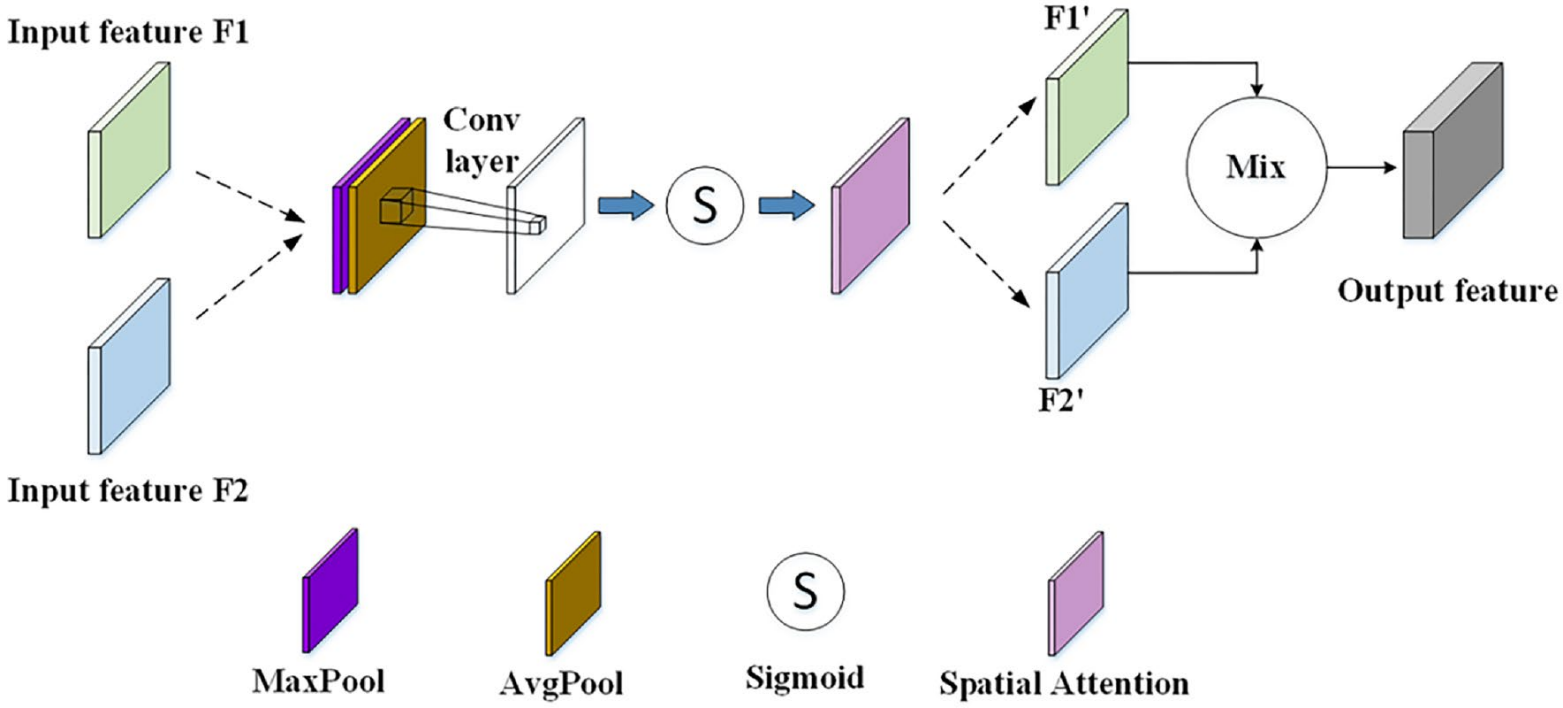
Spatial attention network architecture diagram.

The main architecture of the spatial attention module is shown in Fig. 4. Where the green module F1 and the blue module F2 on the left side represent the input feature maps. The dark purple module denotes the feature map after global maximum pooling and the brown module denotes the feature map after global average pooling. The white module denotes the feature map after convolutional layer, the s symbol denotes the Sigmoid function, the mauve module denotes the spatial attention weights, and the green module F1' and blue module F2' on the right side denote the feature maps obtained by multiplying with the spatial attention weights. The 2D image is passed through the multi-scale network to get the feature map F1, and through the U-Net network to get the feature map F2, F1 and F2 have the same size (64*64*3) and are used as inputs to the spatial attention module. The input feature maps F1 and F2 are first subjected to global maximum pooling and global average pooling in the channel dimension as a way to obtain the maximum and average values of each spatial location, and two 64*64*1 feature maps are obtained. These two pooling results are then spliced in the channel dimension to obtain the 64*64*2 feature map. The weights of each spatial location are then learned by a convolutional layer and a Sigmoid function. Finally, the weights are applied to each spatial location on the feature map to produce feature maps F1' and F2' with enhanced spatial importance.F1' and F2' are spliced in the channel dimension as the final output. F1' and F2' are spliced in the dimensions of the channel as the final output. The use of spatial attention networks enables the model to be more focused on key locations in the input data, which enhances the extraction of meaningful features and improves the performance of the model.

## Experiments and results

### Experimental details

In order to verify the feasibility of the proposed method, we made sufficient preparation for data collection. First, we conducted a detailed study on the working principle and composition structure of the ZPW-2000A track circuit. During the study, we found that when one track circuit segment fails, it may affect two neighboring track circuits. Therefore, we decided to take the faulty section and the two neighboring sections as the object of data collection (the main track and the small track of the faulty section, the main track and the small track of the rear section, and the main track of the front section). The small track of the forward sector is not affected and therefore no data is collected. In the actual work of railroad transportation, monitoring equipment usually collects data at a frequency of once a second, and when a fault occurs it usually lasts for a period of time and will directly affect the fluctuation of the main track voltage and the small track voltage, and most of the faults will be completed within two minutes of the fault. Faults in track circuits are diverse and similar, and the voltage fluctuations may have only small differences between faults, and critical fault voltage characteristics may be lost when data are collected at large intervals. Therefore, we collect the voltage data of the main and small track circuits of three adjacent track circuits at a frequency of once a second for a period of two minutes, including three main track circuits and two small track circuits, totaling 600 voltage features. The smaller the frequency of sample collection, the more detailed the features of the collected samples will be, which will also improve the performance of the model for fault diagnosis. We collected and organized real fault data from a normally operating train station, including normal state and six fault types, and the fault information is shown in Table 1. The collected real fault dataset contains 1400 fault samples and has a size of 3.02 M. The experimental test can show that using smaller dataset can reduce the training cost and training time, but too small dataset may lead to insufficient data fitting and affect the experimental results. By comparing the experimental results, the smaller dataset can't give full play to the performance of the fault diagnosis model, therefore, we use the collected complete dataset for the experiments without reducing the data and size.

**Table 1:** Track circuit fault information.

Fault location	Label	Sample characteristics
None	0	600
Tuning unit	1	600
Matching transformer	2	600
Attenuator	3	600
Rail	4	600
Compensation capaciatance C6	5	600
Compensation capaciatance C10	6	600

Seven fault types are included in the dataset, including normal state and six faults, and the fault locations are shown in Table 1. It contains the equipment that is prone to faults from the sending end to the receiving end of the rail circuit. Different equipment faults result in fluctuations in the main track voltage and small track voltage of the rail circuit. Some of these faults have similarities in voltage fluctuations, which makes diagnosis difficult.

To train the proposed method, we resized all transformed GASF images to 64*64*3 and set the batch size for training to 50. In addition, we trained the network for a total of 10 epochs and used the SGD optimizer to accelerate training, with a learning rate set to 0.01. All experiments were conducted using the PyTorch framework with a GPU. To fully utilize the functions of different networks in the model, it is necessary to set the parameters of the model. First, analyze the characteristics and size of the experimental data. Then, summarize the rules of parameter setting based on experience and literature review. Finally, adjust the parameters through multiple experiments to obtain the best parameters. The parameter settings for the multiscale convolutional network are shown in Table 2, for the U-Net network in Table 3, and for the spatial attention network in Table 4.

**Table 2:** Parameters of multi-scale convolutional networks.

Name	Channels	Kernel size	Input size	Output size
Multiscale Convolutional Neural Network:				
Conv-1	16	3*3	64*64*3	64*64*16
Conv-2	16	3*3	64*64*16	64*64*16
Cat				64*64*32
Res Block-1	32	3*3	64*64*32	64*64*32
Conv-3	16	5*5	64*64*32	64*64*16
Cat				64*64*48
Res Block-2	48	3*3	64*64*48	64*64*48
Conv-4	16	7*7	64*64*48	64*64*16
Cat				64*64*64
Res Block-3	64	3*3	64*64*64	64*64*64
Conv-5	16		64*64*64	64*64*16
Cat				64*64*80
Res Block-4	80	3*3	64*64*80	64*64*80
Conv-6	16	1*1	64*64*80	64*64*16
Conv-7	3	3*3	64*64*16	64*64*3

**Table 3:** U-Net network parameters.

Name	Channels	Kernel size	Input size	Output size
Downsample				
Conv-1	64	3*3	64*64*3	64*64*64
Conv-2	64	3*3	64*64*64	32*32*64
Conv-3	128	3*3	32*32*64	32*32*128
Conv-4	128	3*3	32*32*128	16*16*128
Conv-5	256	3*3	16*16*128	16*16*256
Conv-6	256	3*3	16*16*256	8*8*256
Conv-7	512	3*3	8*8*256	8*8*512
Conv-8	512	3*3	8*8*512	4*4*512
Conv-9	1024	3*3	4*4*512	4*4*1024
Upsample				
Conv-10	512	1*1	4*4*1024	8*8*512
Cat				8*8*1024
Conv-11	512	3*3	8*8*1024	8*8*512
Conv-12	256	1*1	8*8*512	16*16*256
Cat				16*16*512
Conv-13	256	3*3	16*16*512	16*16*256
Conv-14	128	1*1	16*16*256	32*32*128
Cat				32*32*256
Conv-15	128	3*3	32*32*256	32*32*128
Conv-16	64	1*1	32*32*128	64*64*64
Cat				64*64*128
Conv-17	64	3*3	64*64*128	64*64*64
Conv-18	3	3*3	64*64*64	64*64*3

**Table 4:** Parameters of the spatial attention module.

Name	Channels	Kernel size	Input size	Output size
Spatial attention module				
Avg-polling			64*64*3	64*64*1
Max-polling			64*64*3	64*64*1
Cat				64*64*2
Conv-1	1	7*7	64*64*2	64*64*1

### Results and analysis

We employed Accuracy, Precision, Recall, and F1-score to verify the model.

4 $$ Accuracy = \frac{TP + TN}{{TP + FP + FN + TN}} $$

5 $$ Precision = \frac{TP}{{TP + FP}} $$

6 $$ Recall = \frac{TP}{{TP + FN}} $$

7 $$ F1 = \frac{2 \times Precision \times Recall}{{Precision + Recall}} $$

where TP, FP, FN, and TN representing the number of true positive, false positive, false negative, and true negative outcomes, respectively.

**Figure 5:**
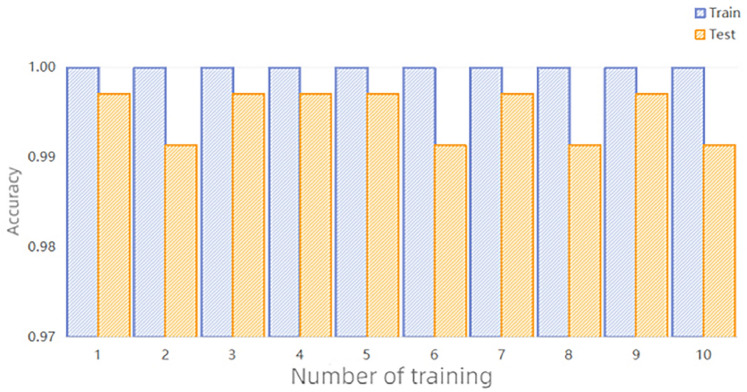
Accuracy of 10 training.

**Figure 6:**
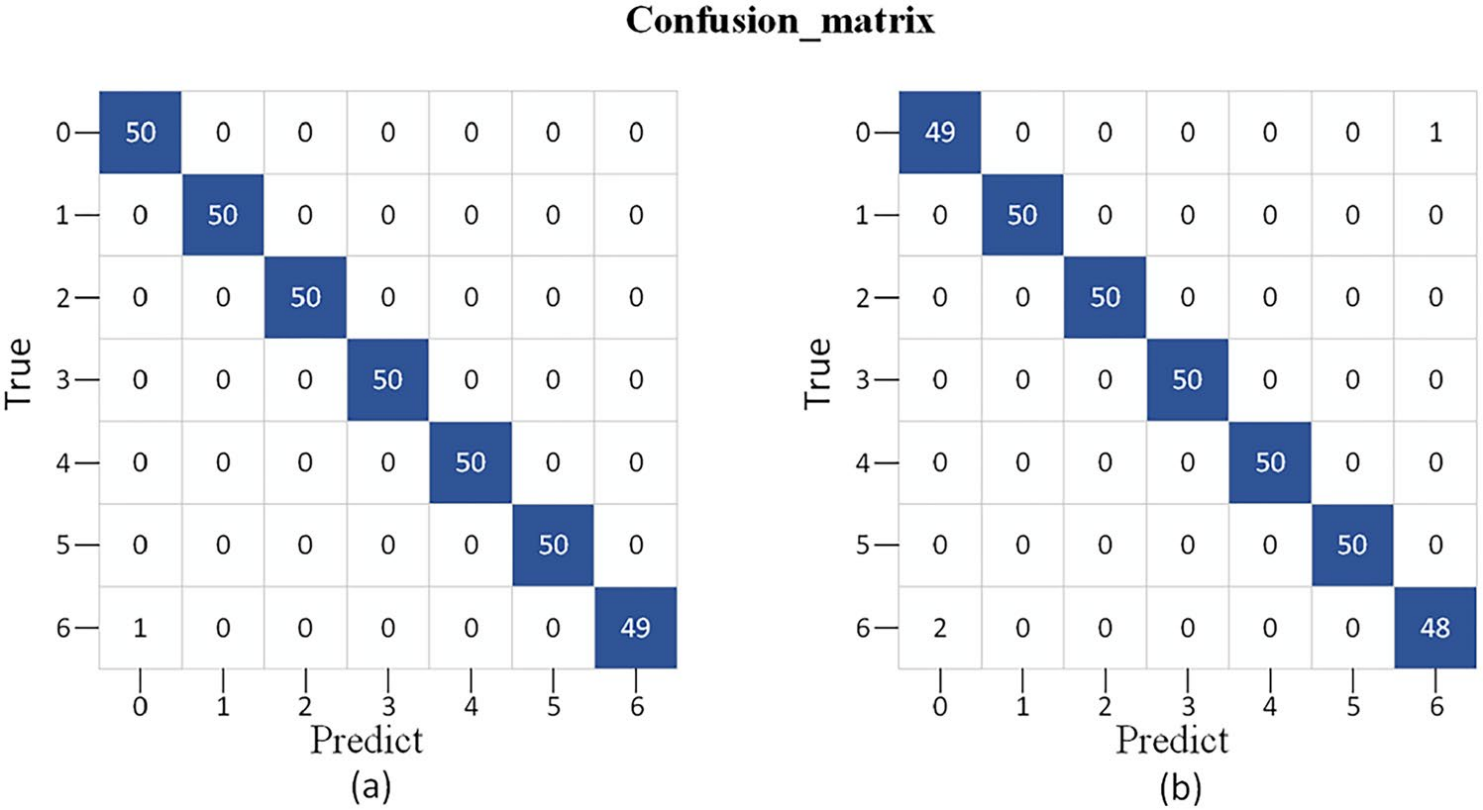
Confusion matrix (**a**) is the maximum accuracy (**b**) is the minimum accuracy.

We conducted ten repeated experiments to avoid experimental randomness. Figure 5 shows the accuracy of the ten experiments, with the test set accuracy exceeding 99%. Table 5 shows the average Precision, Recall, and F1-score for the ten experiments conducted by MSAN. The average accuracy of MSAN is 99.36%. Figure 6 shows the confusion matrix of the highest and lowest accuracies of MSAN (99.71% and 99.14%, respectively). The reason for the lower accuracy in the ten experiments is the misclassification between fault 0 and fault 6. The main reason for this phenomenon is the similarity between the data of fault 0 and fault 6, where there are individual faults with high data similarity, which makes it difficult to distinguish between them. Secondly, the experiments in multiple tests produce different results with small differences, which is caused by two reasons. One is that the experiments are conducted under the same hardware and environment, and the results of the model training are also affected due to the fact that the load conditions of the computers may be slightly different. The second is that the SGD optimization algorithm is used in the experiments, and the gradient of each iteration is random, which also leads to different paths for parameter updating and ultimately affects the performance of the model. However, the overall fault diagnosis results validate the ability of MSAN to effectively detect various track circuit faults.

**Table 5:** Track circuit fault diagnosis results.

Labels	Precision (%)	Recall (%)	F1-score (%)
0	99.02	99.80	99.40
1	99.41	99.80	99.60
2	99.41	99.80	99.60
3	99.60	99.40	99.50
4	99.61	99.60	99.60
5	99.59	99.00	99.29
6	99.80	99.00	99.39
Accuracy	99.36

### The t-distributed stochastic neighbor embedding (t-SNE)

**Figure 7:**
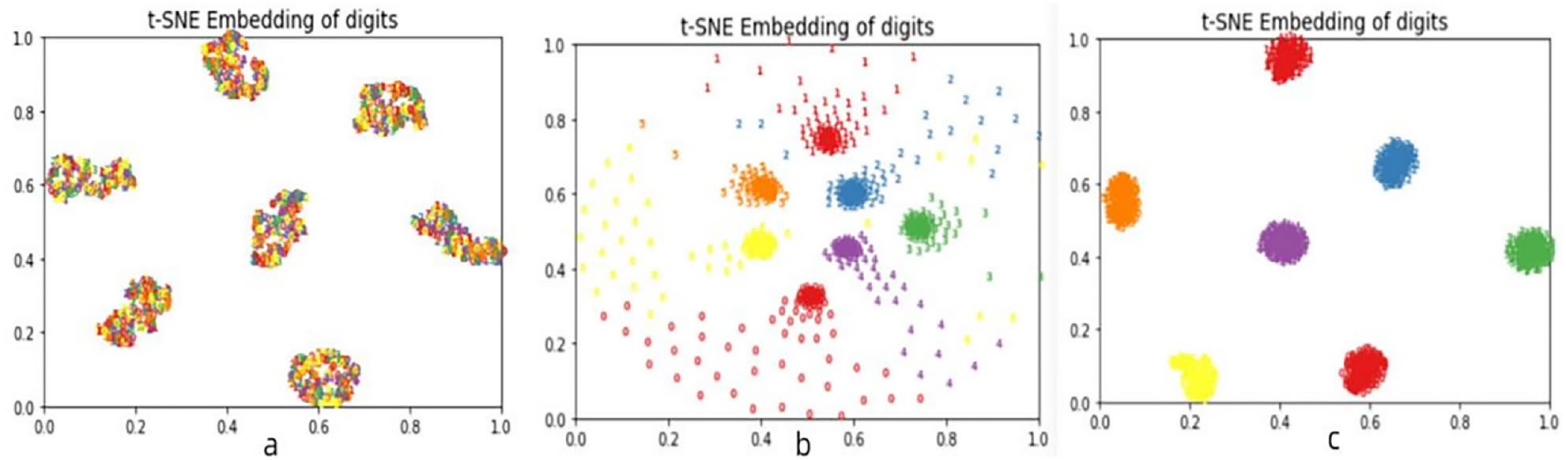
Feature visualization of different modules of MSAN.

t-SNE (t-Distributed Stochastic Neighbor Embedding) is a common data dimensionality reduction and visualization algorithm that can reduce the high-dimensional feature representation in deep learning networks to two or three dimensions, allowing us to better understand and analyze the process of feature extraction in neural networks through visualization. Figure 7 shows the visualization of feature extraction from different modules in MSAN. Panel a shows the visualization of one-dimensional raw data, panel b shows the visualization of features after multi-scale convolution and U-Net module, and panel c shows the visualization of features after spatial attention network. It can be seen that the model's classification performance gradually improves through different modules, which validates the feasibility of this method.

### Ablation study

In order to verify the rationality and interpretability of the proposed MSAN, we conducted ablation experiments on different network structures. In order to ensure the reliability of the experiments, the network parameters used in the experiments were basically the same. To verify the effectiveness of the proposed network structure, we kept the network input unchanged and changed the network structure.

Table 6 shows the accuracy rate and training time rate of different model structures. The training time rate is the ratio of the training time of different models to the training time of the overall model we designed, and from the table, we can see that the network structure we designed for multi-scale fusion not only has high accuracy but also spends the least time.Our proposed method adds multi-scale convolutional network and spatial attention network to U-Net network, we perform ablation experiments on different modules in the proposed method and verify the effect of different modules on the overall performance. The U-Net network in the diagnostic model is first labeled as Module A, the multiscale convolutional network as Module B, and the spatial attention network as Module C. Then the test is performed by deleting the different modules, and the results of the ablation experiments are shown in Table 3. Where the accuracy obtained using the full model was 99.36% and the training time of this model was used as the standard time to evaluate the other modules. After deleting module B and module C in the diagnostic model, module A is tested, and the accuracy of the test is 98.98%, and the training time is 1.86 times the standard time; after deleting module C, module A + B is tested, and the accuracy obtained from the test is 92%, and the training time is 1.23 times the standard time; after deleting module B in the diagnostic model, module A + C is tested, and the accuracy is 99.14% and the training time is 1.56 times the standard time . Therefore, it can be concluded that the deletion of module B has less impact on the accuracy and mainly affects the training time of the model; the diagnostic accuracy of the model is more affected by the deletion of module C.

**Table 6:** Ablation study.

Model	Accuracy	Training Time Rate
A + B + C(ours)	99.36%	100%
A + C	99.14%	156%
A + B	92.00%	123%
A	98.98%	186%

### Comparison with other methods

Most fault diagnosis methods use an end-to-end approach to directly input 1-dimensional raw data. The method proposed in this paper converts 1-dimensional time series data into 2-dimensional images before entering the network for training. In order to ensure the reliability of the experiments, we repeated the experiments using the same dataset with the current classification methods that perform well in the field of fault diagnosis, and compared them with the method used in this paper. The comparison results are shown in Table 7.

**Table 7:** Comparison results of different methods.

Method	Accuracy (%)	Precision (%)	Recall (%)	F1-score (%)
CNN^6^	72.00	72.00	71.97	72.02
SE-MSCNN^7^	88.85	93.74	88.85	91.22
CNN-LSTM^8^	96.28	97.05	96.28	96.67
MSD-CNN^10^	96.86	97.42	96.85	97.17
LSTM^14^	85.71	85.71	78.57	82.02
MSAN(ours)	99.36	99.49	99.48	99.48

From the table, it can be seen that the performance of the proposed method in this paper is higher than other fault diagnosis methods. From the references of the compared diagnostic methods, it can be seen that these methods are able to achieve good results, but they did not achieve good results in our experiments. The reason for this problem is most likely due to the fact that the dataset we used is not generalized. The dataset we used is the main and small track voltages including the faulty track circuit and the two neighboring track circuits. And the frequency of data collection is once a second within two minutes. In the compared references, the dataset used is mostly current data of one track circuit and the frequency of data collection is different. These differences may be the most critical reason for this phenomenon. There are two main reasons why the method proposed in this paper can achieve good results compared to other methods, the first is that most of the methods for the processing of the data are used to normalize or directly do not deal with, we will be the original one-dimensional data through the GAF method into a two-dimensional picture, the transformed picture preserves both the original data information and the temporal correlation of the data, which does not result in the loss of feature information. And it can give full play to the advantages of convolutional networks in processing image data and improve the performance of the model. The second point is that our proposed method fully extracts the feature information of different scales and depths in the data, and mines more useful information, and increases the attention to the key areas through the spatial attention mechanism, which further improves the performance of the model.

## Discussion

In a real railroad system, the ability to acquire and process data in real time in any environment needs to be ensured to ensure that the data used for model training is accurate and reliable, and the existing microcomputer monitoring technology can meet this requirement. A picture conversion device can be installed after the microcomputer monitoring system to convert real-time data into two-dimensional pictures. The fault diagnosis model can be installed after the picture conversion equipment. The railroad system is a large and complex system with a large number of railroad lines, the amount of data collected during fault diagnosis is large, and a system that meets the computational requirements is needed for computation if the fault diagnosis results need to be obtained in time. Therefore, it is theoretically feasible to deploy the model in a real railroad infrastructure.

The method proposed in this paper is applicable to ZPW-2000A track circuit fault diagnosis, and also has certain applicability to the fault diagnosis of different railroad systems. The method proposed in this paper is able to deal with one-dimensional time series data and two-dimensional picture data, and the method is able to make full use of the structure of the convolutional network to deal with the picture data, for the one-dimensional time series data can be transformed into two-dimensional pictures through the GAF method, and then carry out the feature fusion; the two-dimensional picture data can be fused with the feature fusion directly. The feature fusion part extracts features at different scales and performs multiple feature fusions, and finally the attention network is utilized to make the model pay more attention to the key locations of the image. The method has no limitation on the input data and is therefore suitable for fault diagnosis in different scenarios. However, the method performs multiple splicing and convolution operations on different numbers of feature maps in the feature fusion stage, which is more demanding for the equipment performing the test. In the subsequent research to address this issue, we are committed to reducing the complexity of the model and strive to simplify the structure of the model without affecting the performance of the model.

## Conclusion

This paper proposes a new intelligent diagnosis method (MSAN). The data processing module can transform one-dimensional time series data into two-dimensional images while retaining the temporal correlation of the data and fully utilizing the advantages of CNN in processing image data. The feature fusion module first learns signal features of different depths and scales from 2D image data, and then passes through the spatial attention network to obtain spatial feature information. Finally, the classifier module performs fault classification, using the softmax function to convert the neuron output into a probability distribution of track circuit faults (including healthy fault), the accuracy of fault diagnosis was 99.36%. The validity of the proposed MSAN model was verified through ablation experiments, and its superiority was confirmed compared with other fault classification methods. This method can utilize real-time monitoring data and be applied in practical work, suitable for fault diagnosis in different scenarios. However, the computations involved are rather complex, demanding high performance from the equipment. In future research, efforts will be directed towards reducing the complexity of the model and simplifying its structure while enhancing performance.

## Data Availability

The data used to support the findings of this study are available from the corresponding author upon request.
